# The Perspective of Croatian Old Apple Cultivars in Extensive Farming for the Production of Functional Foods

**DOI:** 10.3390/foods10040708

**Published:** 2021-03-26

**Authors:** Boris Duralija, Predrag Putnik, Dora Brdar, Anica Bebek Markovinović, Sandra Zavadlav, Mirian Pateiro, Rubén Domínguez, José M. Lorenzo, Danijela Bursać Kovačević

**Affiliations:** 1 Department of Pomology, Faculty of Agriculture, University of Zagreb, Svetošimunska Cesta 25, 10000 Zagreb, Croatia; bduralija@agr.hr; 2 Department of Food Technology, University North, Trg dr. Žarka Dolinara 1, 48000 Koprivnica, Croatia; 3 Faculty of Food Technology and Biotechnology, University of Zagreb, Pierottijeva 6, 10000 Zagreb, Croatia; brdar.dora@gmail.com (D.B.); abebekmarkovinovic@pbf.hr (A.B.M.); dbursac@pbf.hr (D.B.K.); 4 Department of Food Technology, Karlovac University of Applied Sciences, Trg J. J. Strossmayera 9, 47000 Karlovac, Croatia; sandra.zavadlav@vuka.hr; 5 Centro Tecnológico de la Carne de Galicia, Rúa Galicia Nº 4, Parque Tecnológico de Galicia, San Cibrao das Viñas, 32900 Ourense, Spain; mirianpateiro@ceteca.net (M.P.); rubendominguez@ceteca.net (R.D.); jmlorenzo@ceteca.net (J.M.L.); 6 Área de Tecnología de los Alimentos, Facultad de Ciencias de Ourense, Universidad de Vigo, 32004 Ourense, Spain

**Keywords:** old apple cultivar, biologically active compounds, functional food, agriculture, extensive farming

## Abstract

The Republic of Croatia has a long tradition of fruit growing due to its geographical location, climatic conditions, and high quality of fruit crops, especially apple fruits. Apples can be used for the formulation of functional foods either in processed form (e.g., juice), or as a by-product (e.g., apple pomace). However, there is a growing demand for functional foods derived from ancient and traditional plant sources as they are recognized as a very valuable source of health-promoting bioactive ingredients. Similarly, old apple cultivars (*Malus domestica* Borkh.) are characterized by good morphological and pomological properties, less need for chemicals during cultivation and the higher share of biologically active compounds (BACs) with better sensory acceptability compared to commercial cultivars. However, their nutritional and biological potential is underestimated, as is their ability to be processed into functional food. The importance in preserving old apple cultivars can also be seen in their significance for improving the nutritional composition of other apple cultivars through innovative cultivation strategies, and therefore old local apple cultivars could be of great importance in future breeding programs.

## 1. Introduction

The Republic of Croatia (RC) is a country with a long tradition of fruit production and processing, and the cultivation of old fruit cultivars (e.g., apples) in extensive farming occupies an important place for the economy, agronomy, and public health. Old fruit cultivars in Croatia are a valuable asset and natural heritage, which does not receive enough attention for popularization and processing. This is because the old apple cultivars were mainly grown locally in numerous small orchards and were not involved in scientific breeding programs. Therefore, only the cultivars obtained through systematic breeding have gained more importance on the market. Apple (*Malus domestica* Borkh.) is a fruit species belonging to the rose family (Rosaceae), and is the most commonly grown and consumed fruit in RC. The annual average consumption of apples per household member was 11.5 kg in 2017 [1]. However, there are no accurate data on how many old apple cultivars are grown in RC and how many are consumed fresh and/or for processing.

Apple is a commercially important fruit in RC and the extension of commercial life and reduction of postharvest losses of fruits is mainly based on storage at low temperatures alone or in combination with modified atmospheres (MAs) and controlled atmospheres (CA), which are primarily aimed at reducing total metabolism and thus delaying ripening and senescence. In the last decade, the traditional CA storage regimes (oxygen concentrations above 1 kPa) have been replaced in apples by the use of ultra-low oxygen (ULO) concentrations (<1 kPa). The latest dynamic CA (DCA) allows the use of much lower oxygen concentrations [2]. In addition, the activity of the enzyme polyphenoloxidase, which is the key factor of the enzymatic browning, can be a problem for post-harvesting apples, which is why various pre-treatments such as dipping solutions and non-thermal technologies are suggested [3,4].

Apple fruit quality includes a large group of external and internal characteristics. External fruit quality includes color, shape, size, and absence of defects, while internal quality (which determines eating quality) consists of taste, texture, aroma, nutritional value, sweetness, acidity (contributes to flavor), shelf life, and absence of defects [5]. For instance, after harvesting and before transportation to market or storage, fruits are calibrated by hand or sorting machine based on weight, fruit size, quality, and color characteristics. In addition, apple is a climacteric fruit and requires an increase in respiration rate and ethylene production to trigger the ripening process in an autocatalytic reaction [6]. Ripening is a complex process involving many factors, including hormonal control, which regulates biochemical and physiological changes that determine the final organoleptic and nutritional characteristics of the fruit [7]. Fruit ripening and senescence led to quality degradation and decrease in fruit firmness due to the degradation of pectin, cellulose, and hemicellulose under the action of enzymes such as pectin methylesterase (PME), polygalacturonase (PG), pectin lyase (PL), cellulase, and β-glucosidase (β-Glu) [8].

Looking at the nutritional aspects, apple fruits contain sugars, acids, vitamins, minerals, pectins, and water [9,10]. Additional to their nutritional value, they are also a valuable and easily accessible source of various biologically active compounds (BACs), especially polyphenols such as chlorogenic acid, (+)-catechin, (−)-epicatechin, phloretin, quercetin, and phloridzin [11]. In a recent study, old Croatian apple cultivars were found to have higher overall quality compared to conventional ones [12]. Therefore, old apple cultivars offer great potential for the production of functional products with higher yield of BACs. BACs have been found to contribute to food functionality due to their antioxidant activity that neutralizes free radicals, prevents the formation of new ones in the body, and repairs the cellular damages caused by them [13]. Increased free radicals lead to oxidative stress resulting in oxidative damages, cell death, tissue damages, and various diseases [14,15].

Dietary fiber, found in apples, is also a plant substance with many benefits for human health such as reduction in fat and cholesterol absorption, normalization of digestion, maintenance of intestinal health, and involvement in diabetes control [16,17]. There are also reports of apple pomace, a by-product of the fruit juice industry, being used as raw material for fiber-enriched functional foods [18].

Recently, consumer demand for traditional ‘old’ apples and their products is increasing, as they follow the trend of consuming natural foods, without added pesticides and additives. Unfortunately, old orchards are decaying recently, and therefore, more and older apple cultivars are being lost. As a result, there are a large number of valuable sources of genetic material with desirable fruit morphological-pomological characteristics, for which, some studies have demonstrated better sensory acceptability compared to commercial cultivars [19]. In addition, many old apple cultivars are particularly important due to genes for pests and diseases resistance, drought tolerance, winter hardiness, and unique fruit quality [20].

New knowledge and research on old apple cultivars would strengthen the market for these fruits and their products, prevent the loss of this valuable genetic material, and contribute to greater biodiversity to promote health and overall well-being. Although there is little literature on old apple cultivars in Croatia, the main objective of this article is to give an overview of the perspective of growing old apple cultivars in the Republic of Croatia, with an overview of their biological values and potential in the production of functional foods.

## 2. Apple Production in the Republic of Croatia

In the total fruit production of the Republic of Croatia in 2018, apple stands out as the most economically important fruit species [1]. According to FAOSTAT data (The Food and Agriculture Organization Corporate Statistical Database), apple cultivation in the Republic of Croatia shows an increase from 2016 to 2018, although in the same period, the area of apple orchards decreased [21]. According to Croatian Bureau of Statistics, the total production of apples in 2017 was 56,570 tons on 4838 ha of cultivated area, and the production increased by 63.47% in 2018 [1]. Data with distribution of apple production in the Republic of Croatia for 2017 and 2018 are given in Supplementary Table S1.

In addition, data from Croatian Bureau of Statistics (2020) also show an overall decrease in the area under apple trees in the Republic of Croatia in the period from 2010 to 2019 [1]. At the same time, apple production decreased (Supplementary Table S2). It seems as agricultural production of apples becomes more effective in Croatia, however, overall decrease of areas for cultivation of this cultivar seems discouraging.

According to the age of apple trees, four basic classes can be distinguished in the Republic of Croatia (Supplementary Figure S1). In 2012, apple trees aged 5 to 14 years were predominant (46.30%), followed by young apple trees aged less than 5 years (24.86%), trees aged 15 to 24 years (16.43%), and trees older than 25 years with the lowest share (12.41%). Five years later (2017), the proportion of trees younger than 5 years decreased almost threefold, while apple trees aged 5 to 14 years are still the most represented. The structure of orchards and apple orchards changes significantly over time, which of course also affects fruit quality.

The Croatian Bureau of Statistics also has data on the area of apples by density of plantations in hectares. The density of plantations with less than 400 apple trees/ha occupied an area of 3.84 ha in 2012, and in 2017, there was a significant increase in the area below 361.32 ha. The density of 400 to 1599 apple trees/ha in 2012 was found to be 1207.95 ha of arable land, while in 2017, the area decreased to 1148.15 ha. A drastic decrease in the area planted with a planting density of 1600 to 3199 trees/ha can be seen from 2012 to 2017. In 2012, the area planted at the indicated density was 3541.01 ha and in 2017, it decreased to 1964.04 ha. The planting density of 3200 and more apple trees/ha recorded the largest increase in the area from 45.84 ha in 2012 to 985.58 ha in 2017 [1]. So, data imply that Croatian growers prefer smaller orchards for production of apples.

Various apple cultivars are grown in the Republic of Croatia, and their areas of cultivation change significantly over time. The most common apple cultivars in Europe are ‘Golden Delicious’, ‘Gala’, ‘Jonagold’, ‘Red Delicious’, ‘Idared’, ‘Elstar’, ‘Granny Smith’, ‘Braeburn’, ‘Fuji’, ‘Jonathan’, and ‘Pink Lady’ and many of these cultivars are grown in Croatia [22]. The total area of apple tree plantations decreased from 2012 to 2017. In 2017, the largest area was cultivated with Idared (36%), Golden Delicious (16%), and Jonagold (10%), where only Golden Delicious increased plantation for given period. The largest jump in the increase of planted area was recorded by ‘Fuji’ (+137%), followed by 59% increase for ‘Gala’ (Supplementary Table S3). These cultivars are valued by the consumers and the demand for them has increased, and so has the area under cultivation. On the other hand, for Elstar and Florina, the largest decrease in cultivation was noticed. Interestingly, it was noticed a drastic decrease in the area planted with ‘Idared’ in 2017. However, as it was earlier planted on the largest area as compared to other cultivars, the relative decrease in cultivation was only around 19%.

Old apple cultivars in extensive farming are becoming more popular and are increasingly in demand on the market. A very important branch of production in the Republic of Croatia is the supply of apples to its own market. Consumers in developing markets demand information about the origin of food and the impacts of production on the environment and food safety. With consumer preference for domestic apples that are produced and processed in a sustainable manner, a further increase in the production and processing of old apple cultivars is expected.

### 2.1. Old Apple Cultivars in Extensive Farming in the Republic of Croatia

Old apple cultivars are the natural and cultural heritage of the Republic of Croatia, as well as a valuable source of genetic diversity. Cultivars that have adapted to local agro-ecological growing conditions and have grown there for a long period of time are recognized as the basis for market-oriented, organic apple cultivation [23]. The main requirement of traders and consumers is high fruit quality [24]. Sensory characteristics (appearance, texture, taste, and aroma), nutritional value, chemical composition, mechanical properties, and functional ingredients define fruit quality [25]. Harvesting timing and post-harvest treatments such as storage, handling methods, and transportation to markets, as well as handling in retail also determine apple quality [23]. According to Westwood [26], the primary quality-related parameters are sugar and acid content, color, firmness, texture, juiciness, taste, nutritional value, absence of disease or insects, and general appearance [26]. Old cultivars show good resistance to biotic and abiotic stress factors [27], and are characterized by different morphological and pomological characteristics compared to commercial apple cultivars.

**Figure 1:**
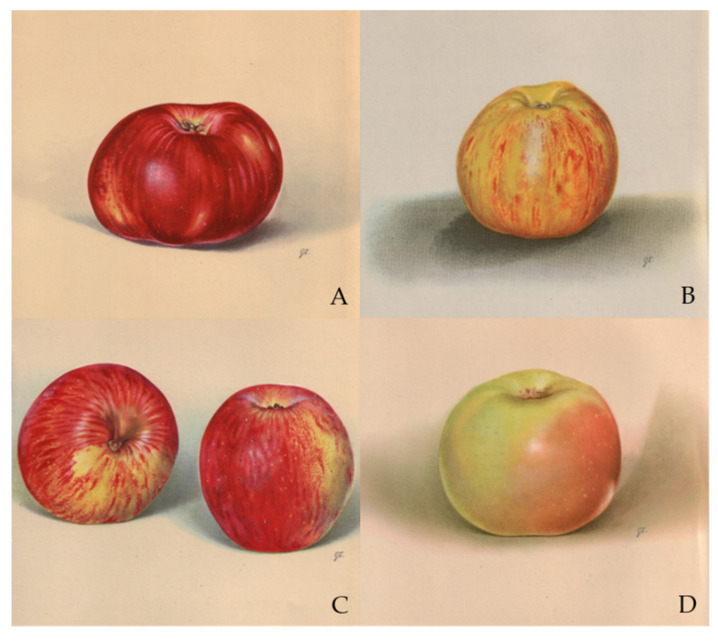
Old apple cultivars ‘Roter Pogatscher’ (‘Božićnica’) (**A**), ‘Blumen Calvill’ (‘Grafenštajnka’) (**B**), ‘Großer Rheinischer Bohnapfel’ (‘Bobovec’) (**C**), and ‘Grüner Stettiner’ (‘Zeleni štetinec’) (**D**) (This is a painting by a Croatian artist Greta Turković).

Skendrović Babojelić et al. [28] stated that fruits do not always look as the first-class fruit [29], but they are distinguished by their different fullness of taste and especially expressed aroma. Janjić [23] describes the morphological and physical characteristics (mass, height, and width of apple fruit) of the following old cultivars: ‘Roter Pogatscher’ (‘Božićnica’) (Figure 1A), ‘Blumen Calvill’ Grafenštajnka’) (Figure 1B), ‘Großer Rheinischer Bohnapfel’ (‘Bobovec’) (Figure 1C), ‘Grüner Stettiner’ (‘Zeleni štetinec’) (Figure 1D), ‘Weißer Winterkalvill’ (‘Bijeli zimski kalvil’), ‘London Pippin’ (‘London peping’), ‘Yellow Bellflower’ (‘Lijepocvjetka’), and ‘Reinette de Champagne’ (‘Šampanjka’).

‘Weißer Winterkalvill’ (‘Bijeli zimski kalvil’) is characterized by medium-thick to thick fruits of irregular asymmetrical shape, delicate pale green to yellow peel, and greenish to white flesh with a slightly sour taste. This cultivar has the largest mass and the largest average width, compared to other analyzed cultivars, but it is susceptible to diseases and pests and more demanding for cultivation. ‘Großer Rheinischer Bohnapfel’ (‘Bobovec’) shows abundant yield with medium-large to large fruits of smooth green peel with red streaks, flat in shape, and resistant to shocks [30]. This cultivar is suitable for processing due to a juicy flesh with good resistant to browning.

‘London Pippin’ is described as a heavy fruit of medium size with the highest height, compared to the other analyzed cultivars, with a medium-thick straw-yellow peel [23]. The flesh is also yellow and has a desirable texture. The fruits of this cultivar are sensitive to transport and many losses are documented due to rot of the fruit during storage [30]. ‘Yellow Bellflower’ (‘Lijepocvjetka’) is an old cultivar of medium-sized to large cone-shaped fruit with a straw-yellow peel on the sunny side. The fruit is firm at harvest time, later becoming gentle and sensitive, and brown spots appear on the otherwise white-yellowish flesh.

‘Reinette de Champagne’ (‘Šampanjka’) is characterized by medium-sized flattened fruits with the smallest height with a very delicate peel of greenish-yellow to white-yellowish color. The flesh of this cultivar is white and has a very fine texture that is not degraded by storage. ‘Grüner Stettiner’ (‘Zeleni štetinec’) also has large and flattened fruits, a thin green peel, and a light-yellow flesh with a sweet-sour taste without a special aroma. The plant cells of flesh have a profound capacity to regenerate therefore the fruits of this cultivar may be stored late spring.

When considering the overall fruit quality, it can be assumed that the desirable fruit quality might be obtained by the cultivar ‘Großer Rheinischer Bohnapfel’ (‘Bobovec’) with the highest share of healthy fruits (92%) after storage for 160 days in a refrigerator at normal atmosphere. It is followed by ‘Yellow Bellflower’ (‘Lijepocvjetka’) cultivar (77%), ‘Grüner Stettiner’ (‘Zeleni štetinec’) (71%), ‘Reinette de Champagne’ (‘Šampanjka’) (63%), ‘London Pippin’ (‘London peping’) (60%), and ‘Weißer Winterkalvill’ (‘Bijeli zimski kalvil’) (43%), while ‘Roter Pogatscher’ (‘Božićnica’) cultivar counts only 40% of healthy fruits, thus it is the cultivar with the highest proportion of rotten fruit [23]. For cultivars with a higher percentage of rotten fruits, it is recommended to perform storage in the refrigerator for a shorter time in order to prevent spoilage and degradation of bioactive compounds [23].

Vujević described the morphological characteristics of two other old cultivars from the Bjelovar-Bilogora County: ‘Reinette du Canada’ (‘Kanada’) and ‘Goldparmäne’ (‘Zlatna zimska parmenka’) [31]. The fruits of ‘Reinette du Canada’ are characterized by a moderately flattened shape of reddish peel with a basic green color. The fruits are sensitive to storage, but have a high tolerance to transport. The firm flesh of the fruit is yellowish-white in color and has a sweet-sour taste. ‘Goldparmäne’ is another old cultivar with a moderately flattened shape of the fruit with firm consistency. The average weight, as well as the height and width of the fruit of this cultivar are higher than reported values for ‘Reinette du Canada’. The peel is dark red, while the flesh is yellowish white and sweet in taste.

Skenderović Babojelić et al. [28] conducted a physico-chemical analysis of the old apple cultivars (‘Großer Rheinischer Bohnapfel’—Bobovec, ‘Roter Pogatscher’—Božićnica, ‘Yellow Bellflower’—Lijepocvjetka, and ‘Goldparmäne’—Zlatna zimska parmenka) grown in the territory of the Topusko, the municipality in Sisak-Moslavina County (continental Croatia). Authors analyzed the color parameters via colorimeter, the hardness of the fruit with a manual penetrometer, the proportion of soluble dry matter with a refractometer, and the proportion of total acids by titration method. The highest fruit hardness was observed in the cultivars ‘Großer Rheinischer Bohnapfel’ and ‘Citronka’, slightly lower in the cultivar ‘Goldparmäne’, and the lowest in the cultivars ‘Roter Pogatscher’ and ‘Yellow Bellflower’. ‘Citronka’ cultivar exhibited the highest starch degradation index while the lowest value was found in cultivar ‘Großer Rheinischer Bohnapfel’. During the process of fruit ripening, the starch is intensively decomposed into simple sugars [32]. In cultivars ‘Goldparmäne’ and ‘Citronka’, the highest values of soluble dry matter were determined, and the cultivar ‘Roter Pogatscher’ was the fruit with the lowest values. The content of soluble dry matter increases during fruit ripening and storage, and it is a good indicator of the sugar content in the apple fruit [33]. The highest number of total acids was determined in the cultivars: ‘Yellow Bellflower’ and ‘Goldparmäne’, which were identified as the most acid apples, followed by ‘Roter Pogatscher’, ‘Großer Rheinischer Bohnapfel’, and ‘Citronka’. Acids have an important role in fruits, as they can slow down the harmful effects of bacteria, degradation of ingredients, and spoilage. In the process of ripening, sugar accumulates, and total acids are broken down, resulting in fruits that become more and more harmonious in taste. In the conclusion, all investigated cultivars were different due to all evaluated parameters, although all cultivars were of good quality and acceptable physiochemical properties. Authors suggested that it is important to preserve old apple cultivars as due to the withering of old trees and a possibility of losing an important source of genetic material, and consequently reduce assortment of apple cultivars on the markets [28].

Hoehn et al. [28] found that consumers are primarily concerned on the fruit size and color when choosing an apple, and that other properties are less important to them. Old cultivars are more resistant to diseases and pests, do not require a large number of pesticide application, as well as intensive care, so they are easily adaptable to organic farming, although they can rarely be purchased in stores. Despite some uniform pomological properties have been observed in old cultivars as compared to commercial apple cultivars, they possess great potential for organic and ecological fruit growing that is becoming increasingly popular. This is well aligned with increasing consumers’ awareness about old cultivars with their specific morphological properties and valuable bioactive compounds, vitamins and minerals without the risks of harmful effects of applied pesticides.

### 2.2. Sustainable Technologies for Cultivation, Selection, and Preservation of Old Apple Cultivars

Mainly old apple cultivars grown in Croatia are found in rural areas in small orchards, and they are very well adapted to local environmental conditions. The old cultivars are grafted on the more vigorous seedling rootstocks and develop a much larger root system and a higher tree crown compared to the modern cultivars grafted on the less vigorous rootstocks. This improves resistance to climatic damage such as higher resistance to drought, longer shelf life, and better anchorage under windy conditions [34]. Compared to intensive industrial apple production in Croatia, where a small number (less than 20) of cultivars exist in plantations (Table 1), more than 50 different genotypes are present in cultivation on small farms. The displacement of old and locally well-adapted cultivars by a few widely used modern cultivars has led to a dramatic loss of genetic diversity in orchards [35]. Many of the well-known, international dominant apple cultivars are closely related, whereas old cultivars were collected over a longer period of time and are more diverse [36]. One of the main objectives of apple breeding networks is the re-diversification of cultivar use and the widespread application of genetic analysis to produce separate cultivar lists for each region containing those best suited for environmentally friendly production [37].

Table 1 lists some factors that have an important influence on apple fruit traits. Understanding the factors that can influence fruit characteristics is critical to obtain a high-quality raw material for processing. Global apple production faces many challenges such as reduced biodiversity in orchards, climate change, water scarcity and pollution, harmful chemical residues in fruits, use of non-renewable resources, less nutrients in modern apple fruits, etc. In the future, all attained knowledge should be taken into consideration and the new apple orchard must be planned for more sustainable farming practices. Some old cultivars have many advantages for sustainable cultivation, as they are better adapted on the environmental factors in the growing area and require less use of energy and chemicals.

The most representative and widely accepted criteria and objectives of sustainable agriculture were adopted in the Den Bosch Declaration [53]. Briefly, sustainable agriculture can be defined as the efficient production of safe, high quality agricultural products in a manner that protects the natural environment, improves the social and economic conditions of farmers, their employees, and local communities, and ensures the health and welfare of all managed species [54]. The guiding principle of sustainable agricultural production is to make the most efficient use of available resources and production potential while minimizing adverse impacts on soils, water, air, and biota [27].

FAO (The Food and Agriculture Organization) sets out the five key principles that balance the social, economic, and environmental dimensions of sustainability: (i) improving the efficiency of resource use; (ii) conserving, protecting, and enhancing natural ecosystems; (iii) protecting and enhancing rural livelihoods and social well-being; (iv) strengthening the resilience of people, communities, and ecosystems; and (v) promoting good stewardship of both natural and human systems [27].

In the temperate zone, cultivated apple is an important fruit crop with an annual production of over 80 million tons on nearly 5 million hectares [55]. The old apple cultivars have a long history of cultivation and grow in different locations in Croatia. Some of the most important conditions for successful growth and development are temperature, water, light, suitable soil, and proper management system. Compared to modern orchard cultivation practices with old cultivars, there are many differences (Supplementary Table S4).

In apple production, there is increasing interest in developing more environmentally friendly production, either through integrated production or organic management [56]. To ensure the sustainability of fruit production, farmers are being pushed to adopt farming practices that may reduce yield and profitability [57]. Management improvements and technologies adopted should be site-specific in terms of soil, landscape, and climate, and should take into account the specific types of apple orchards and farms [58].

## 3. Apple as a Functional Food

### 3.1. Apples and Apple Products as a Source of Functional Ingredients

Consumers today demand value-added foods (e.g., functional foods) that are sustainably produced and processed, considered safe, fresh, natural, and have important nutritional value [59]. Therefore, as the most widely consumed temperate fruit species in the world, apple has great potential for the production of functional foods. The chemical composition of apple fruit is extremely complex as it is rich in many nutrients and thus considered as a major source of phytochemicals in the human diet [60]. To that end, Scalbert et al. [61] discovered several phenolic compounds in apple, being (+)-catechin and (−)-epicatechin (flavan-3-ols or flavanols), phloridzin (dihydrocalconglycosides), quercetins (flavonols), cyanidin-3-*O*-galactosides (anthocyanins), hydroxycinnamic acids, and hydroxycinnamic acid (chlorogenic acid and *p*-coumaroylquinic acid). The phenolic profile and antioxidant capacity for different parts of the apple in different cultivars are given in Table 2. The presence of these and other bioactive components has also been reported for apple products and by-products (Table 3).

Phenolic compounds are not evenly distributed in apple fruit, i.e., certain components of phenolic compounds are present in certain parts of the fruit while very few or none are present in remaining parts. For this reason, many researchers propose to use apple peels or apple cores as a by-product or waste for the subsequent extraction of phenolic compounds. Tsao et al. [62] reported five times more polyphenols in apple peel than in apple flesh. The authors attributed this difference to the defensive role of the peel in protecting the fruit from harmful UV light and environmental pathogens. McGhie et al. [63] found that about 46% of apple polyphenols are found in the peel, i.e., all flavonols (quercetin derivatives) are determined only in the peel, which would explain the greater antioxidant capacity of the peel (Table 2). Chinnici et al. [27] concluded that flavonols, flavanols, and procyanidins are most contribute to the antioxidant activity of the apple peel, about 90% of the total activity calculated.

Among the processed apple products, apple juice as well as apple cider are the most popular [64]. Due to the different localization of polyphenols in the pulp, apple juice and fresh apple differ in their composition. During juice production, only some of the phenolic compounds are extracted into the juice, while most of the polyphenols remain in the solid residue after the juice is pressed [65]. The solid residue consists of peels and cores, and therefore phenolic compounds such as quercetin glycosides and dihydrocalcones are present in small amounts in apple juice [27].

Regarding juice quality, consumers prefer clear apple juices that do not lose many valuable components with high antioxidant potential during the production process [66]. During the clarification process, mainly (−)-epicatechin and procyanidins are mostly removed. Lee et al. [67] observed a significant contribution of flavonoids such as quercetin, (−)-epicatechin, and procyanidin B2 to the total antioxidant activity of apples, in contrast to the antioxidant contribution of vitamin C. The antioxidant activity (AOA) of juices obtained by pressing was 10% of that of fresh ‘Joanagold’ apples, while the AOA of clear juices showed only 3% of that of fresh apples, with a 50% decrease in chlorogenic acid and a 3% decrease in catechins. After processing and subsequent storage of apple juice at room temperature (25 °C) for nine months, a significant loss of antioxidant polyphenols was observed. Despite the fact that polyphenols in apple juices were more stable than vitamin C, significant losses of quercetin (60%) and procyanidins (100%) were observed [27,68]. Van der Sluis et al. [69] showed that elevated temperature during apple juice storage negatively affected the stability of polyphenolic antioxidants, with quercetin glycosides and epicatechins being the most heat sensitive, and phloridzin and chlorogenic acid the most stable.

Apple fruit pigmentation is controlled by the relative amounts of anthocyanins, chlorophylls, and carotenoids. Anthocyanins and carotenoids have been shown to have potent antioxidant and anticancer properties [70]. Since they have potential beneficial effects on human health, new cultivars with improved pigmentation are being developed, such as red-fleshed apples with increased anthocyanin concentration.

Apples, apple peels, and apple flesh are also an important source of triterpene compounds (1.635–3.173 mg g^−1^ dry weight), being ursolic acid the most significant constituent (72.1–81.2%), followed by oleanolic acid, corosolic acid, and betulinic acid [71]. Dashbaldan et al. [72] determined the profile of neutral triterpenoids, triterpenic acids, steroids, and esters in apple cultivar ‘Antonovka’, and observed significant changes in triterpenoid contents during fruit growth and ripening. The importance of triterpenoids found in apples was demonstrated by their high potential of anti-proliferative and anticancer activities [72].

The by-products of apple fruit processing contain considerable amounts of fibers, and therefore have attracted the attention of scientists and industry due to the increasing waste utilization [73]. There are about 2.21 g of dietary fiber in 100 g of apple fruit. Insoluble dietary fiber (cellulose and hemicellulose) accounts for 70%, while soluble dietary fiber (pectin) accounts for 30% [74]. Sun-Waterhouse et al. [75] isolated dietary fibers with high pectin polysaccharide content and phenolic antioxidants from ‘Granny Smith’, which could be a new type of functional ingredients. Phenolic compounds can bind to cellulose and pectin through covalent bonds via esters or carbon from the cell wall to form insoluble bound phenols.

### 3.2. Bioactive Potential of Old Apple Cultivars from Extensive Farming

Old apple cultivars were more appreciated by consumers according to the tested sensory properties and nutritional characteristics as compared to the commercial ‘Golden Delicious’ [85]. Old Portuguese apples have been shown to have higher bioactive potential, containing more fiber, protein, sugar, β-carotene, vitamin E, magnesium, and polyphenolic compounds compared to the commercial vs. such as ‘Fuji’, ‘Gala Galaxy’, ‘Golden’, ‘Reineta Parda’, and ‘Starking’ [27].

Old apple cultivars have qualitatively identified the same subgroups of polyphenols as the commercial cultivars [39,86,87]. Further results of other studies indicated that the proportions of phenolic compounds were equal in old and new apple cultivars [88]. Iacopini et al. [89] investigated an antiradical potential of old vs. commercial Italian apple cultivars. It was found, that the higher total phenolic content as well as total flavonoids were in two of the four old varieties analyzed, as compared with commercial cultivars. When observing individual phenolic compounds, the HPLC qualitative pattern was similar in all the examined cultivars although higher values was confirmed for old cultivars. Moreover, old cultivars exhibited a higher antioxidant activity compared to the commercial cultivars.

Preti and Tarola [40] recently evaluated fourteen ancient apple cultivars grown in northeast Italy to investigate their nutraceutical properties comprising polyphenols, antioxidant capacity, as well as four major minerals (Na, K, Mg, Ca) in comparison to commercial cultivars. All the analyses were performed on apple peel and pulp, separately. Peel samples showed a significantly higher contents in phenolic compounds with respect to pulp, almost threefold than in pulp for ancient cultivars and quadrupled for commercial cultivars. Dihydroclacones phloridzin and phloretin were mostly found in peel, with higher proportions in ancient apples as compared to commercial counterparts. The importance of phloridzin was described with influence on lower susceptibility of apple fruit to the most important diseases, thus providing resistance to the common apple pathogens such as *Venturia inaequalis* and *Erwinia amylovora* [90].

Though old apple cultivars have shown higher polyphenol content, it is important to note that environmental conditions may have an important impact on the amount of polyphenols [12]. The polyphenol profile could also be affected by different farming methods, i.e., agricultural practice such as conventional, integrated, or organic [91]. The color of apples, and therefore the proportion of pigments, can be influenced by the geographical location of orchards [92]. Volz and McGhie [93] concluded that differences in polyphenol content between cultivars might be as the result of genetic variability. Carbone et al. [76] reported that the genotype of old apple cultivars could have a positive impacts on the content of bioactive compounds, and for this reason, old cultivars were presented as an important source of genes for future breeding programs. Due to the favorable differences in the characteristics of old cultivars, which have developed through their long-term growth in Croatia, these cultivars should be preserved for the future and further popularization among growers, producers, and consumers [27].

Jakobek and Barron [39] analyzed the peel and flesh of the old apple cultivars from the area of Slavonia (Continental Croatia) with the aim of highlighting and preserving the biological diversity of apple cultivars with the greater bioactive potential. Using the high-performance liquid chromatography (HPLC) with a diode-array detector (DAD, they determined the content and composition of polyphenolic compounds, and the results indicated that the following cultivars were the richest in polyphenols: ‘Zimnjara’, ‘Lještarka’, and ‘Adamova Zvijezda’. The authors noted that the cultivars differ according to their content of individual polyphenolic groups, some of them are richer in the phenolic acids, and others in flavan-3-ols, i.e., cultivars in which phenolic acids occupy a larger proportion, while others that contain a smaller amount of flavan-3-ols, and vice versa. Lastly, it can be concluded that it is possible to classify old apple cultivars based on the predominant proportion of flavan-3-ols or phenolic acids [79].

According to the results for the investigated commercial apple cultivars, the content and composition of polyphenols in old apple cultivars also significantly varies depending on the parts of the fruit. Jakobek et al. [87] showed that the concentration of phenolic acids was significantly higher in the peel than in the flesh for all samples of old apple cultivars, which is consistent with research that was already available [63,87,94,95,96]. Authors investigated thirteen old apple cultivars ‘Ljubeničarka’, ‘Astrahan’, ‘Crvenka’, ‘Kardinal’, ‘Kraljevina’, ‘Ružica’, ‘Pisanica’, ‘Petrovka’, ‘Slavonska Srčika’, ‘Bjeličnik’, ‘Ledenara’, ‘Štegerova’, and ‘Jaje’ grown in the region Slavonia (Mihaljevci, near Požega) in Continental Croatia.

Proanthocyanidins are proven as the most abundant analyzed bioactive compounds that made up between 70–90% of total polyphenolic compounds in apples [62,80,88,95,97]. Jakobek et al. [87] firstly analyzed oligomeric pronatocyanidins in the old local apple cultivars from Southeastern European region after acid hydrolysis in the presence of the organic compound fluoroglucinol (C_6_H_6_O_3_). In this way, the authors were able to obtain information about the constituent units of proanthocyanidins, as well as their locations within the complex molecular structure, using HPLC coupled to UV–vis detection and ultra-high performance liquid chromatography with quadrupole time-of-flight (UPLC-Q-TOF). In the majority of previous studies, the proportion of oligomeric proanthocyanidins is often neglected because there was no conversion to subunits, but only monomers, dimers, and trimers are considered [62,98,99,100,101]. For this reason, Jakobek et al. [87] could not correctly compare the obtained results with the results found in the previous studies. They proved that this method is effective for the characterization and quantification of proanthocyanidins in apple fruits.

Phenolic acids were the second most abundant subgroup in apples, with proportions of 6–25% in the flesh and 1–10% in the peel. The highest values were detected in the samples of the cv. ‘Slavonska srčika’, followed by ‘Kardinal’, ‘Astrahan’, ‘Kraljevina’ (red apples), and ‘Bjeličnik’ (green or yellow apples). Flavonols were found mainly in all peel samples, in a proportion of 1–13% of the total content of phenolic compounds in the peel. Much smaller amounts were found in the flesh, which is consistent with the results of research conducted for other apple cultivars [62,95,97]. ‘Astrahan’ and ‘Slavonska srčika’ showed the highest proportions of flavonols in their composition, while the lowest values were found in the cv. ‘Štegerova’ [87]. Dihydrochalcones were observed in peel samples, from 1–10% of the total polyphenolic content, and a smaller share were found in the flesh of the fruit. The cultivars with the highest concentration of dihydrochalcones in the peel were ‘Petrovka’ and ‘Slavonska srčika’, and the smallest amount was detected in the cultivar ‘Štegerova’. Anthocyanins were found only in the samples of red-peeled apples, in a proportion of 1–7% of the total polyphenol content. The lowest value was detected in the samples of the cultivar ‘Slavonska srčika’, which was found to be the richest in the remaining phenolic compounds. The highest amounts of anthocyanins were determined in the samples of the cultivar ‘Ljubeničarka’. In conclusion, ‘Slavonska Srčika’ was highlighted as the cultivar with the largest proportion of all phenolic compounds in total phenolic content, except anthocyanins. The cultivar ‘Ljubeničarka’ had a reddish-colored flesh, which is unusual for apples, because the cultivar contained anthocyanins in the flesh, as well as in the peel. Scientists predict that ‘Ljubeničarka’ could be important in future apple growing programs due to its attractive red color and high bioactive potential [27].

Jakobek et al. [12] reported that the most of the analyzed old cultivars from the towns of Donji Miholjac and Gornji Tkalec and the village of Rude contained higher amounts of polyphenols in the flesh and in the peel as compared to the commercial apple cultivars. This is precisely the quality characteristic with bioactive potential that should be emphasized for old cultivars. Some cultivars can be distinguished by a higher proportion of polyphenols in the peel, as is the case with ‘Pisanike’, ‘Adamove zvijezde’, ‘Zelenike’, and ‘Kanada’. Cultivars that can be characterized by the higher amounts of polyphenols identified in the flesh were ‘Božićnica 2’, ‘Boskop’, ‘Zimnjara’, and ‘Crveni boskop’. All identified polyphenols from five different subgroups, have already been reported in the literature [74,102,103]. By comparing the total amounts of identified subgroups of polyphenols in flesh samples, it was noticed that phenolic acids were the most prevalent. Moreover, flavonols gave the main contribution to the total amounts of polyphenols in apple peel samples.

Apples are also a natural source of important dihydrochalcones [104,105]. Dihydrochalcones are a specific subgroup of polyphenols, found mainly in apples with the potential to lower blood glucose levels, which may be useful in diabetes management [106,107]. Almost all old cultivars were found to had a higher proportion of dihydrochalcones compared to the commercial cultivars which is also one of the important reasons for considering more extensive apple growth [87].

In further analysis of polyphenols in old cultivars, it is important to point out that the flesh of the fruit contributes more to the absorption of polyphenols in the body of a person who consumes an apple. The peel only makes up about 10% of the entire fruit and is not always consumed with it. For this reason, cultivars that contain more polyphenols in the flesh might be a better source of polyphenols. Although the peel does not contribute to polyphenolic intake to the same extent as the pulp, it contains important polyphenolic groups such as quercetin derivatives and a high concentration of other polyphenols [87]. Differences between genotypes accounted for 46–97% of the total difference in the concentration of total polyphenols and polyphenolic groups in flesh and peel [93]. Flavonols in the peel protect the fruit from UV radiation, although they are more sensitive to environmental changes due to their sensitivity to light and temperature changes [93]. It is suggested that the high content of flavonols (quercetin derivatives) in the peel of the analyzed old apple cultivars is due to environmental conditions and is very likely to change depending on climatic conditions [87]. The content of other identified polyphenols, especially in the pulp, could result from genetic variability [87]. Table 4 provides an overview of research results on the main polyphenolic subgroups of old apple cultivars grown in Croatia.

Lanzerstorfer et al. [108] investigated the content of minerals, phosphates, and trace elements, as well as the content of polyphenols in apple juices from old apple cultivars. They found large differences between the investigated cultivars regarding the content of the mentioned elements. The authors have concluded that the old apple cultivars can serve as functional apple products with emphasis on desirable health effects.

Apples were shown to have the potential to cause allergic reactions [109]. In this regard, existing studies have shown that old cultivars are better tolerated by individuals with apple intolerance than new cultivars due to their high polyphenol contents [27]. Vegro et al. [110] demonstrated that the genetic material of old cultivars is less allergenic [110]. Barreira et al. [111] suggested in their study that phenolic compounds from old apple cultivars, which are more than those present in the commercial cultivars, can be used in dermal formulations due to the many useful properties such as antioxidant or antimicrobial activity. Ikumi et al. [112] also proposed oral antidiabetic agents based on phloridzin conjugates.

## 4. The Influence of Apples on Health

The health effects of apples are influenced by the availability of the bioactive compounds contained in apple and by their absorption and metabolism in the human body. The bioavailability of polyphenols depends on the amount of phenolic compounds that are released from solid foods in the body and can pass the intestinal barrier [113]. Fruits and vegetables are naturally composed of hydrated cells with phenolic compounds in cell vacuoles that are only weakly bound to the cell wall [114]. The mechanical action of digestion causes these cells to burst and allows the release of the phenolic compounds, while at the same time, the acidic environment of the stomach and the alkaline environment of the intestine facilitate the release of polyphenols close to the cell wall. Wruss et al. [115] explained the reason for the inconsistent results of clinical studies on the health benefits of apple and individual flavonoids by the specific pharmacokinetics that occur due to differences in small intestinal length, intestinal microbiota, or genetic factors of each individual. These variations in each human organism have significant effects on polyphenol metabolism. The current research available on the health benefits of apples is summarized below (Supplementary Figure S2).

For instance, a synergistic interaction of polyphenol rich foods and the gut microbiota has been demonstrated. Microorganisms in the colon can release polyphenols from the fibrous environment and break them down into phenolic acids, while polyphenols are able to stimulate the growth of beneficial bacterial species and inhibit the growth of pathogenic species [116]. The effect of polyphenols in the human body also depends on the genetic characteristics of the individual. In addition, there is growing evidence that the bioavailability and bioefficiency of polyphenols are influenced by the environment in which the polyphenols are found, i.e., the other bioactive components of the apple, as well as by the dose of polyphenols ingested. Jakobek [117] demonstrated a synergistic interaction between dietary fiber and flavonoids, which has a positive effect on human health, as well as the interaction of lipids and polyphenols which reduces the process of fat absorption, and thus has positive effects on health. Dietary fiber possesses a protective role in the treatment and prevention of certain diseases such as intestinal diseases [118]. The beneficial effects of apples consumption on vascular function and blood pressure prevention have been demonstrated, lowering blood lipids, reducing inflammation and preventing hyperglycemia [27].

The protective effect of apples and other fruits against cardiovascular diseases are attributed to the high content of polyphenols and their specific composition. Apples make an important contribution to the intake of macronutrients associated with the prevention of cardiovascular disease. There is a significant association between intake of more dietary flavonoids and the reduction of mortality, especially coronary mortality in women [119]. Knekt et al. [119] observed an association of increased quercetin intake with lower coronary heart disease mortality. Arts et al. [120] showed in a prospective study of postmenopausal Iowa women that reduced mortality from coronary heart disease has favored the intakes of the flavonoids (+)-catechin and (−)-epicatechin. Recent studies indicated a 46% reduction risk of cardiovascular disease mortality in elderly men by (−)-epicatechin intake, with 28% of the total (−)-epicatechin intake coming from apples. An increasing number of studies reported a lower incidence of coronary heart disease and cardiovascular disease in subjects consuming large amounts of dietary fibers [121]. Consumption of 120 g of apple flesh with 80 g of peels provided a higher intake of quercetin, (−)-epicatechin, and other flavonoids, compared to the control group that consumed only the flesh of the apple. Higher flavonoid consumption resulted in lower systolic blood pressure and pulse pressure in a randomized controlled trial of 30 healthy men and women [122]. The study showed an acute increase in nitric oxide, which causes smooth muscle relaxation, leading to dilatation of blood vessels and a lowering of a blood pressure, i.e., vasodilation. When studying the effect of apples on cholesterol levels, consumption of three apples per day resulted in a 5–8% reduction in total cholesterol, while consumption of apple juice (375–720 mL) had no effect on plasma cholesterol levels and had a deleterious effect on plasma triglyceride levels due to its high fructose content [123]. In postmenopausal women, significantly lower total serum cholesterol levels were found after 6 months of consumption of dried apples compared to 6 months of consumption of prunes [124]. In addition, a study of apple polyphenol consumption (1500 mg daily for four weeks) reflected that total cholesterol was reduced by 4.5% in 48 men and women with high cholesterol levels [125]. Phenolic compounds from apples have also been shown to increase lipoprotein lipase activity, thereby lowering blood cholesterol levels [126]. In human digestion, pectin can potentially lower plasma lipid levels by binding to cholesterol in the gastrointestinal tract [127]. Although the effect of pectin on lowering cholesterol has been reported, the relatively low pectin content in apples suggests the presence of other apple components that may have an effect.

Inflammatory processes are present in a variety of human diseases, and there is evidence that polyphenols have anti-inflammatory effects [128]. In a study of 8335 adults in the United States, apple consumption was inversely related to C-reactive protein (CRP) levels. CRP serves as an inflammatory marker and is therefore an important indicator of inflammation [129]. Chai et al. [124] observed that consumption of dried apples over a 12-month period reduced CRP levels by 32%, but this did not reach a statistically significant difference compared to the control sample. In a meta-analysis of studies on increasing fiber consumption in humans, six out of seven studies reported significant reductions in CRP levels. Prebiotic fibers have been shown to affect intestinal permeability, reducing the absorption of lipopolysaccharide, an endotoxin that releases Gram-negative bacteria that elicit a strong immune response in humans [9].

Western dietary habits are considered to cause disease, while a diet rich in fruits and vegetables is associated with risk reduction. The increase in type 2 diabetes worldwide is a cause for public health concern, since type 2 diabetes may increase the risks of cardiovascular disease [130]. Apples have been highlighted as an important dietary component that has the potential to reduce the growing prevalence of type 2 diabetes. Consumption of more than one apple per day is associated with a significant reduction in type 2 diabetes risk (by 28%) compared with non-apple consumption [131]. The evidence that certain types of polyphenols may reduce the risk of type 2 diabetes comes from a study of 2915 participants. This study found that each 2.5-fold increase in flavonol intake was associated with a 26% lower incidence of type 2 diabetes [132].

Additionally, higher intake of soluble fibers has a beneficial effect on reducing the development of type 2 diabetes. Dietary fiber has been attributed to a beneficial effect on weight loss, and thus on the treatment of obesity [16]. Consumption of two apples per day for two weeks resulted in a significant increase in human intestinal bifidobacteria and fecal acetic acid, i.e., a positive effect on the health of the gut microbiota [133].

Dysbiosis or disturbance of the intestinal microflora is a term used to describe the disturbance of the natural balance of microorganisms in the digestive tract. The disturbance results from a decrease in the proportion of beneficial microorganisms, an overgrowth of potentially harmful microorganisms or a decrease in overall microbiological diversity [134]. The effect of quercetin supplementation on the suppression of dysbiosis of the intestinal microbiota, caused by a diet high in sucrose and fat has been demonstrated. Quercetin reduced the ratio of *Firmicutes* and *Bacteroidetes* (markers of intestinal health) and inhibited the growth of *Erysipelotrichaceus*, *Bacillus*, and *Eubacterium cylindroides* bacteria (bacterial species associated with diet-induced obesity) [135]. Phloretin, found in apples, has been shown to act as an inhibitor of pathogenic biofilm by *Escherichia coli* production and as an anti-inflammatory agent in inflammatory bowel disease [136]. A recent study confirmed a protective effect of phloridzin on antioxidant stress, DNA damage, and apoptosis in H_2_O_2_^−^ induced HepG2 cells, therefore many studies suggested that phloridzin can be used in the production of functional foods [137]. In conclusion, the health effects of apples are diverse and have been shown to be beneficial, so the recorded evidence points to the potential of apples for the production of functional products.

## 5. The Perspective and Development of Apple-Based Functional Products Behind Functional Ingredients

Functional foods are defined as industrially processed foods or unprocessed natural foods that have been shown to have beneficial health effects beyond basic nutritional value when consumed regularly as part of a varied diet [138]. For this reason, functional food is an increasingly popular term in social and scientific circles. Food manufactures are also investing in the development of industrially processed foods that may have additional health benefits for consumers. It is very important that clinical (randomized, double-blind, and placebo-controlled) trials are conducted before drawing conclusions about the health benefits of functional foods [139]. Clinical studies and experimental evidence will provide an answer as to which food ingredient is functionally effective for the human body and to what extent. In addition, for a food to be labeled functional, it must be confirmed in intervention tests that it complies with the regulations of the country. Some of the regulations that functional foods must comply with are the European Food Safety Authority (EFSA) in European Union and the Food and Drug Administration (FDA) in the United States [140,141,142]. Functional foods must be safe and freely available to consumers without the need for a prescription from a physician [143]. It is necessary to emphasize that functional foods are not medicine; they will not prevent or cure diseases, as various internal and external factors play a crucial role in the occurrence of the disease [144,145]. To some extent, consumers regulate the trends in the food industry and their growing preference for safe, fresh, and natural foods that have health benefits may explain the importance of the adaptability of the food industry and investment in the production of functional products. Therefore, the production of functional products is expected to increase rapidly across the globe.

Apple juice is commonly mixed with another liquid ingredients (extract, tea, beverage, etc.) to formulate an ideal functional food formulation with improved physicochemical properties, nutritional characteristics, and sensory acceptability [146]. A recent study aimed to formulate a functional cake based on apple pomace flour (powder) as a substitute for wheat and rice flour to produce a gluten free product suitable for celiac patients [147]. Apple pomace flour had lower protein content (1.25%) and higher fiber content (56%) compared to wheat and rice flours. The proximate composition of the flour showed that the content of total phenols in apple pomace flour was 4 times higher than in wheat and 7 times higher than in rice flour. The authors concluded that by replacing 100% of the flour with apple pomace, the physical and sensory properties could be satisfactory according to consumer expectations. Although cakes with apple pomace flour had a harder texture and lower specific volume, their general acceptance in terms of good smell and taste made them highly desirable products [27].

The whole unripe apples ‘Golden Delicious’ without non-edible parts (seeds and peduncle) were used to obtain apple flour by convection drying at 50 °C for 4 h [148]. Grounded dried pieces were used for making spaghetti-type pasta by replacing 50% of durum wheat semolina with oat bran in order to monitor cooking quality, digestibility, antioxidant, nutritional, and texture characteristics. The cooked pasta with apple flour showed the higher content of total phenols and scavenging capacity in comparison with the control samples. Based on the good retention of bioactive compounds and antioxidant capacity after the cooking process of the noodles, the authors concluded that apple flour could be considered as a sustainable food ingredient for the development of a functional food [148]. In addition, apple pomace water extract could also be considered as a very valuable ingredient for the supplementation or development of fortified foods such as functional yogurt formulations [149]. Yogurt fortified with apple pomace extract showed improved fiber content and antioxidant properties compared to plain yogurt. In this regard, probiotic yogurt inoculated with *Lactobacillus acidophilus*, *Streptococcus thermophilus*, and *Bifidobacterium bifidum* to which 3% apple pomace flour was added showed optimal functional properties in terms bioactivity and sensory characteristics compared to control samples [27].

The use of innovative technologies such as high hydrostatic pressure processing (HHPP) can also help to improve the functional properties of the by-products of the apple ‘Golden Delicious’ [150]. De la Peña Armada et al. [150] found that HHPP under 200 MPa for 15 min was sufficient to improve the solubilization of cell wall components such as pectins, increase the content of soluble carbohydrates that could act as prebiotics, and maintain the content of total phenols compared to untreated samples. The use of HHPP to modify the chemical properties of apple by-products even makes them more suitable for industrial application in the production of functional foods.

Pulsed electric fields (PEF) is another innovative approach used to improve the functional quality of apples without affecting their physicochemical properties [151]. Important changes in phenolic profile and quality properties of raw apples (firmness, color properties, soluble solids content, pH, titratable acidity) were induced by PEF treatment as a function of specific energy and time after treatment. Lower energy (0.01 kJ kg^−1^) had a positive effect on bioactive compound content, while higher energy (1.8 and 7.3 kJ kg^−1^) resulted in irreversible quality changes (texture and color). This PEF effect was explained as a response of apple tissues to oxidative stress, which led to accumulation of bioactive compounds after treatment. Another possible explanation is related to matrix changes and increased extractability of bioactive compounds during their determination [27].

## 6. Conclusions

Food origins and safety are increasingly becoming critical factors in the selection of fruit cultivars for direct consumption, as well as for processing into products. Old apple cultivars with a long history of cultivation on the territory in the Republic of Croatia have been shown to generally require fewer agricultural inputs compared to commercial apple cultivars, but are nevertheless unsuitable for large-scale distribution.

However, their growth, yield, and desirable fruit quality characteristics make them more attractive for both, as an important gene source for apple breeding programs and as commercial cultivars for the domestic supermarket channel and touristic markets.

Their potential for processing into functional foods is supported by evidence of health benefits due to higher levels of polyphenolic compounds and antioxidant capacity as compared to commercial cultivars. Improving yields and efficiency, developing market products, improving knowledge, experience and entrepreneurship, strengthening cooperation, improving product safety and quality e.g., by implementing certification programs, and agro-logistics are the main priorities for improving the competitiveness of old apple cultivars and their functional products on the Croatian and EU markets.

## Figures and Tables

**Table 1:** Factors affecting apple fruit quality in sustainable cultivation.

Factor	Effect on Fruit Quality Characteristics	Ref.
Cultivar	Mean fruit mass, shape, firmness, SSC, TA, color	[38]
	Polyphenols (flavonols, dihydrochalcones, flavanols, phenolic acids, anthocyanins)	[39]
	Antioxidant capacity, mineral content	[40]
Rootstock	Mean fruit mass, firmness, SSC	[41]
Interstock	Firmness, SSC, starch content	[42]
Tree age	Firmness, flavor, color	[43]
Environment	Mean fruit mass, firmness, SSC, TA, ascorbic acid	[44]
	Color, anthocyanins	[45]
Plant densities	Soluble solids content, organic acids, sugars	[46]
Training and pruning	SSC, TA, mineral contents	[47]
Production system	Mean fruit mass, mineral content	[48]
Yield	Mean fruit mass, SSC, color	[49]
Agro-techniques	Mean fruit mass, firmness, sugars	[50]
	Mean fruit mass, firmness, SSC, TA, antioxidant capacity	[51]
Harvest time	Mean fruit mass, firmness, ethylene concentration	[52]

**Table 2:** The phenolic profile and antioxidant capacity for different parts of the apple in different cultivars.

Cultivar (Sample)	Phenolic Compund	Concentration (mg 100 g^−1^)	Antioxidant Capacity *	Conclusion Remarks	Reference
*Fuji cl. Kiku8* (peel)	chlorogenic acid	8.96 ± 1.22	EC_50_ = 17.34	- The content of phenolic compounds was influenced by the clone and the part of fruit (peel vs. mesocarp). - With no impact of cultivar, higher antioxidant capacity and total phenols were found in peel samples as compared to mesocarp samples. Therefore, peels accounted for the increased index of antiradical capacity in comparison with mesocarp. - The highest antioxidant capacity was determined in peels of Fuji and mesocarp of Golden cl. B. - Quercetin-glucoside was determined only in apple mesocarp, while in peels was not present. - Golden cl. B was found as the sample with highest content of phenolic compounds in peels and mesocarp. - The highest content of (+)-catechin was determined in peels of Fuji cl. Kiku8 and Golden cl. B. - The highest content of (−)-epicatechin was detected in peels of Fuji cl. Kiku8.	[76]
	(+)-catechin	12.91 ± 0.5			
	(−)-epicatechin	9.94 ± 0.76			
	phloretin glucoside	1.04 ± 0.25			
	quercetin glucoside	14.92 ± 2.32			
*Braeburn. Hillwell* (peel)	chlorogenic acid	2.82 ± 0.38	EC_50_ = 22.67		
	(+)-catechin	9.81 ± 0.73			
	(−)-epicatechin	5.12 ± 0.65			
	phloretin glucoside	0.77 ± 0.4			
	quercetin glucoside	11.54 ± 5.27			
*Golden cl. B* (peel)	chlorogenic acid	8.64 ± 2.12	EC_50_ = 18.667		
	(+)-catechin	12.18 ± 1.11			
	(−)-epicatechin	6.03 ± 1.11			
	phloretin glucoside	1.05 ± 0.72			
	quercetin glucoside	23.51 ± 2 78			
*Fuji cl. Kiku8* (mesocarp)	chlorogenic acid	5.76 ± 1.22	EC_50_ = 39.236		
	(+)-catechin	0.98 ± 0.31			
	(−)-epicatechin	1.75 ± 0.64			
	phloretin glucoside	1.09 ± 0.06			
*Braeburn. Hillwell* (mesocarp)	chlorogenic acid	5.32 ± 1.33	EC_50_ = 58.48		
	(+)-catechin	0.91 ± 0.18			
	(−)-epicatechin	1.91 ± 0.33			
	phloretin glucoside	0.75 ± 0.09			
*Golden cl. B* (mesocarp)	chlorogenic acid	7.52 ± 0.9	EC_50_ = 26.596		
	(+)-catechin	2.20 ± 1.34			
	(−)-epicatechin	1.46 ± 0.17			
	phloretin glucoside	1.00 ± 0.11			
*Rome Beauty* (mesocarp)	Total phenols	93.0 ± 4.1	ND	- The total phenols of the peels were significantly higher than the mesocarp and mesocarp + peel values within all cultivars, while the total phenols of the mesocarp were not significantly lower than the mesocarp + peels contents. - Within all apple cultivars, the peels contained the highest content of flavonoids, followed by the mesocarp + peel and the mesocarp. - The total antioxidant capacity of the peels was higher than that of the flesh or flesh + peel for all cultivars.	[77]
*Idared* (mesocarp)		75.7 ± 4.0	ND		
*Cortland* (mesocarp)		103.2 ± 12.3	EC_50_ = 103.9 ± 16.5		
*Golden Delicious* (mesocarp)		97.7 ± 8.9	EC_50_ = 155.3 ± 11.7		
*Rome Beauty* (peel and mesocarp)		159.0 ± 15.1	EC_50_= 26.5 ± 0.3		
*Idared* (peel and mesocarp)		120.1 ± 15.0	EC_50_= 125.1 ± 58.8		
*Cortland* (peel and mesocarp)		119.0 ± 14.9	EC_50_= 74.1 ± 4.0		
*Golden Delicious* (peel and mesocarp)		129.7 ± 9.7	EC_50_= 107.7 ± 22.7		
*Rome Beauty* (peel)		500.2 ± 13.7	EC_50_= 12.4 ± 0.4		
*Idared* (peel)		588.9 ± 83.02	EC_50_= 13.6 ± 0.2		
*Cortland* (peel)		388.5 ± 82.4	EC_50_= 15.7 ± 0.3		
*Golden Delicious* (peel)		309.1 ± 32.1	EC_50_= 20.2 ± 0.7		
*Idared* (peel)	procyanidin B2	23.47 ± 0.01	6.02 ± 0.104 mmol trolox 100 mL^−1^	- In both apple varieties, total phenolic content was greater in the peel, followed by the mesocarp + peel and the mesocarp. - ‘Idared’ apple peel had a higher TPC than ‘Fuji’. - Quercetin was only determined in the peel samples, mostly in the form of glycosides, galactosides, xylosides, arabinosides, rhamnosides, being the rutinosides as the most common. - The highest antioxidant capacity was determined in the peels, followed by the samples of peels and mesocarp, while the lowest values were detected in the mesocarp samples. - Antioxidant capacity in both ABTS and DPPH assays was - positively correlated with total phenolic compounds found in the peel, mesocarp + peel, and mesocarp.	[78]
	phloridzin	4.32 ± 0.13			
	(−)-epicatechin	3.33 ± 0.04			
	chlorogenic acid	2.54 ± 0.06			
	quercetin glucoside	0.56 ± 0.05			
	rutin	3.26 ± 0.06			
	qercetin	0.08 ± 0.001			
*Fuji* (peel)	procyanidin B2	31.41 ± 0.13	5.13 ± 0.23 mmol trolox 100 mL^−1^		
	phloridzin	ND			
	(−)-epicatechin	6.39 ± 0.09			
	chlorogenic acid	ND			
	quercetin glucoside	3.88 ± 0.004			
	rutin	8.33 ± 0.14			
	qercetin	ND			
*Idared* (peel and mesocarp)	procyanidin B2	12.41 ± 0.43	2.46 ± 0.06 mmol trolox 100 mL^−1^		
	phloridzin	1.54 ± 0.09			
	(−)-epicatechin	1.12 ± 0.02			
	chlorogenic acid	9.16 ± 0.13			
	quercetin glucoside	0.3 ± 0.02			
	rutin	2.93 ± 0.05			
*Fuji* (peel and mesocarp)	procyanidin B2	9.48 ± 0.35	2.69 ± 0.08 mmol trolox 100 mL^−1^		
	phloridzin	0.87 ± 0.03			
	(−)-epicatechin	1.70 ± 0.05			
	chlorogenic acid	4.69 ± 0.03			
	quercetin glucoside	0.13 ± 0.01			
	rutin	0.95 ± 0.02			
*Idared* (mesocarp)	procyanidin B2	3.13 ± 0.027	1.67 ± 0.04 mmol trolox 100 mL^−1^		
	phloridzin	0.72 ± 0.02			
	(−)-epicatechin	0.45 ± 0.01			
	chlorogenic acid	8.05 ± 0.08			
	quercetin glucoside	ND			
*Fuji* (mesocarp)	procyanidin B2	0.57 ± 0.01	2.09 ± 0.04 mmol trolox 100 mL^−1^		
	phloridzin	1.16 ± 0.08			
	(−)-epicatechin	3.82 ± 0.03			
	chlorogenic acid	3.13 ± 0.027			
	quercetin glucoside	0.72 ± 0.02			
*104 apple cultivars* (whole fruit)	catechin	1.66	-	- There are large differences between apple cultivars with respect to polyphenol content and profile. - Two major subclasses of polyphenols, flavan-3-ols and phenolic acids, were found as predominant in the apple polyphenol profile. - By calculating the flavan-3-ol to phenolic acid ratio, apple cultivars can be classified into flavan-3-ol rich or phenolic - acid rich.	[79]
	epicatechin	7.72			
	procyanidin B1	2.71			
	procyanidin B2	8.58			
	chlorogenic acid	17.44			
	coumaroylquinic acid	2.18			
	phloridzin	2.38			
	phloretin-xyloglucoside	3.63			
	quercetin-galactoside and quercetin-glucoside	1.45			
	rutin	0.48			
	quercetin-rhamnoside	1.45			
*Renetta*	Total phenols	211.9	-	- Flavanols (catechin and proanthocyanidins) were reported as the major class of polyphenols (71–90%) in red apples, followed by hydroxycinnamates (4–18%), flavonols (1–11%), dihydrochalcones (2–6%), and anthocyanins (1–3%).	[80]
*Red Delicious*		131.1			
*Granny Smith*		121.0			
*Morgendulf*		105.8			
*Golden Delicious*		86.3			
*Royal Gala*		83.9			
*Braeburn*		75.4			
*Fuji*		66.2			

* TPC—Total phenolic content; ABTS—The 2,2-azino-bis(3-ethylbenzothiazoline-6-sulfonic acid) (ABTS•+) radical cation-based assay; DPPH (2,2-diphenyl-1-picryl-hydrazyl-hydrate) scavenging activity; EC50 = mg of tissue on fresh weight basis required to obtain 50% DPPH scavenging; ND = not detected.

**Table 3:** The phenolic profile and antioxidant capacity in apple products and apple by-products.

Sample	Phenolic Compund	Concentration (mg 100 g^−1^)	Antioxidant Capacity *	Conclusion Remarks	Reference
Industrial apple pomace consisted of 3 cultivars: *Fuji*, *Qinguan*, *Granny Smith* (six fractions gradually eluted with aqueous alcohol (20%, 40%, 60%, 80% and 100%)	chlorogenic acid	1.30	43.45 ± 3.45 51.65 ± 9.57 90.96 ± 10.23 71.54 ± 2.41 10.12 ± 2.31 9.68 ± 4.55	- Fraction 3 had the highest phenolic content, while the lowest contents was determined in fractions 5 and 6. - The capacity of scavenging free radicals varied in the order: fraction 3 > fraction 4 > fraction 2 > fraction 1 > fraction 5 > fraction 6. - Fraction 3 had the highest contents of chlorogenic acid, syrigin, procyanidins B2, and quercetin. - None of the nine phenolic compounds detected was found in fractions 5 and 6.	[81]
	syrigin	0.44			
	procyanidins B2	32.31			
	caffeic acid	0.15			
	cinnamic acid	0.40			
	phloridzin	0.18			
	quercetin	23.93			
	hyperin	5.20			
	(−)-epicatechin	ND			
*Limón Montés* (single-cultivar pomaces)	chlorogenic acid	681.5	12.4		
	(−)-epicatechin	161.1			
	phloridzin	587.2			
	quercetin	252.0			
*De Ia Riega* (single-cultivar pomaces)	chlorogenic acid	1415.5	13.5		
	(−)-epicatechin	314.6			
	phloridzin	730.2			
	quercetin	96.0			
M1, M3—48 h M2—10 h M4—36 h (Mixture of Asturian apples, hydraulic press, and different degrees of exposure to air during the pressing process)	chlorogenic acid	586.7	7.6 12.5 10.3	- Eleven different cider apple pomaces (six single-cultivar and five from the cider-making industry) were investigated for phenolic profiles and antioxidant capacity. - The group of single-cultivar pomaces showed higher contents of chlorogenic acid, (−)-epicatechin, procyanidin B2 and dihydrochalcones, whereas the industrial samples revealed higher amounts of up to four unknown compounds, with absorption maxima between 256 and 284 nm. - ‘Meana’ was the cultivar with the lowest amount of trimers and tetramers, and the highest in other flavanols, followed by ‘De la Riega and Carrió’. - Phloridzin was determined as the main dihydrochalcone present in the apple pomaces, followed by phloretin-2’-xyloglucoside. - Chlorogenic acid was the major phenolic acid in all the samples. - Asturian cultivars present higher concentrations of (−)-epicatechin, chlorogenic acid and phloridzin than those observed in the Basque region.	[82]
	(−)-epicatechin	ND			
	phloridzin	302.5			
	quercetin	144.2			
	chlorogenic acid	602.4			
	(−)-epicatechin	287.1			
	phloridzin	594.7			
	quercetin	109.9			
	chlorogenic acid	375.3			
	(−)-epicatechin	ND			
	phloridzin	292.5			
	qercetin	87.1			
G—1.5 h (Mixture of Asturian and foreign apples Pneumatic, Bucher–Guyer type press)	chlorogenic acid	259.8	8.2		
	(−)-epicatechin	167.5			
	phloridzin	451.6			
	quercetin	186.3			
*Cripps Pink* *Golden Delicious* (Minimally processed apples: treated with antibrowning solution (calcium ascorbate)	chlorogenic acid	1.13	12.68 ± 0.26 9.50 ± 0.38 (mmol trolox kg^−1^)	- The application of anti-browning agents did not affect the amount of phenolic compounds, but showed improved antioxidant capacity compared to control samples. - Phenolic compounds were stable while the values of antioxidant capacity decreased during storage.	[83]
	(−)-epicatechin	0.05			
	phloridzin	0.03			
	quercetin-3-galactoside	0.03			
	chlorogenic acid	0.60			
	(−)-epicatechin	0.07			
	phloridzin	ND			
	quercetin-3-galactoside	0.03			
Cloudy juice from ‘*Golden Delicious*’ treated by High Power Ultrasound (HPU) and stored at 4 °C	Total phenols Total flavan-3-ols (0 day)	1.86 ± 0.09 0.53 ± 0.03	5.93 ± 0.2 (mmol trolox g d. w.^−1^)	- HPU significantly decreased phenolic compounds and antioxidant capacity in the samples. - Storage had a significant effect on total phenols, flavan-3-ols and DPPH values, decreasing the values by 89.21%, 82.80%, and 79.51%, respectively.	[84]
	Total phenols Total flavan-3-ols (7th day)	0.40 ± 0.09 0.08 ± 0.03	1.60 ± 0.2 (mmol trolox g d. w.^−1^)		

* DPPH scavenging rate (%); ND = not detected.

**Table 4:** The main polyphenolic subgroups of old apple cultivars grown in Croatia (mg kg^−1^ fresh sample weight).

Cultivar	Sample	Total flavan-3-ols	Total Dihydrochalcones	Total Phenolic Acids	Total Flavonols	Total Anthocyanins	Total Phenols	Reference
*Crvenka*	peel	1179	212	319	964	200	2874	[12]
*Crveni boskop*		316	168	178	644	41	1347	
*Pisanika*		653	195	396	2513	44	3801	
*Lještarka*		542	169	42	1994	64	2811	
*Božićnica 1*		400	222	176	639	42	1479	
*Božićnica 2*		493	267	224	1342	93	2319	
*Coxs orange*		332	32	83	583	46	1076	
*Ivanlija*		287	47	517	1532	17	2400	
*Boskop*		268	287	138	240	12	945	
*Bobovac*		484	80	260	1104	12	1940	
*Slavonska srčika*		102	53	18	359	4	536	
*Kolerova srčika*		1077	133	519	1038	8	2775	
*Batulenka*		305	28	42	552	3	930	
*Gravenstein*		287	20	23	266	9	605	
*Mašanka*		280	45	105	1006	5	1441	
*Kanada*		364	231	161	2316	3	3075	
*Kandil Sinap*		438	79	27	119	/	663	
*Citronka*		99	51	221	458	/	829	
*Zimnjara*		256	232	308	312	/	1108	
*Zlatica*		242	19	51	388	/	700	
*Gloria Mundi*		231	26	51	1376	/	1584	
*Zelenika*		550	54	68	2451	/	3123	
*Krastavka*		225	103	35	209	/	573	
*Adamova zvijezda*		1358	151	547	1486	5	3547	
*Crvenka*	mesocarp	33	9	145	8	/	195	
*Crveni boskop*		56	30	598	20	/	704	
*Pisanika*		56	13	134	18	/	221	
*Lještarka*		23	10	141	17	/	191	
*Božićnica 1*		42	42	423	20	/	527	
*Božićnica 2*		112	48	1058	26	/	1294	
*Coxs orange*		47	18	457	20	/	542	
*Ivanlija*		42	18	400	22	/	482	
*Boskop*		152	68	840	27	/	1087	
*Bobovac*		48	7	134	16	/	205	
*Slavonska srčika*		24	2	56	13	/	95	
*Kolerova srčika*		93	17	325	19	/	454	
*Batulenka*		10	2	55	12	/	79	
*Gravenstein*		48	11	258	14	/	331	
*Kandil Sinap*		140	14	118	13	/	285	
*Citronka*		31	18	226	11	/	286	
*Zimnjara*		75	47	603	16	/	741	
*Zlatica*		9	5	64	13	/	91	
*Mašanka*		9	7	155	17	/	188	
*Kanada*		17	12	128	18	/	175	
*Gloria Mundi*		23	4	107	20	/	154	
*Zelenika*		15	6	72	16	/	109	
*Krastavka*		66	12	137	18	/	233	
*Adamova zvijezda*		48	7	205	16	/	276	
*Ljubeničarka*	mesocarp of red and light red apples	3892.0	53.1	534.4	/	318.8	4798.3	[87]
*Astrahan*		3342.0	67.0	663.6	3.8	/	4076.4	
*Crvenika*		3804.0	138.1	259.0	/	/	4201.1	
*Kardinal*		5482.0	113.3	1011.1	/	/	6606.4	
*Kraljevina*		2978.0	82.0	750.9	2.4	/	3813.3	
*Ružica*		4412.0	60.8	381.1	/	/	4853.9	
*Pisanica*		3228.0	95.8	606.1	2.8	/	3932.7	
*Slavonska Srčika*		5326.0	149.5	1381.3	3.4	/	6860.2	
*Ljubeničarka*	peel of red and light red apples	9984.0	124.0	212.3	199.4	761.0	11,280.7	
*Astrahan*		8386.0	269.4	64.2	1455.8	556.8	11,318.2	
*Crvenika*		7538.0	486.8	34.9	399.4	437.2	8896.3	
*Kardinal*		9694.0	290.2	739.2	427.0	251.8	11402.2	
*Kraljevina*		11,788.0	207.7	393.1	294.4	410.4	13,093.6	
*Ružica*		8948.0	472.0	98.2	279.6	402.2	10,200.0	
*Pisanica*		7122.0	424.2	479.6	397.4	403.0	8826.2	
*Slavonska srčika*		11,062.0	707.2	1190.0	963.6	79.4	14,002.2	
*Petrovka*	mesocarp of green or yellow apples	1844.0	168.2	639.4	/	/	2651.6	
*Bjeličnik*		2450.0	102.1	842.8	/	/	3394.9	
*Ledenara*		3884.0	105.4	376.9	4.4	/	4370.7	
*Štegerova*		3300.0	45.1	508.0	/	/	3853.1	
*Jaje*		3448.0	1.6	579.9	3.0	/	4142.5	
*Petrovka*	peel of green or yellow apples	6124.0	769.3	248.2	251.4	/	7392.9	
*Bjeličnik*		4532.0	418.9	611.7	322.8	/	5885.4	
*Ledenara*		8862.0	388.2	116.0	419.4	/	9785.6	
*Štegerova*		5922.0	74.5	386.2	85.2	/	6467.9	
*Jaje*		5474.0	338.6	199.0	471.4	/	6483.0	

## Supplementary Materials

The following are available online at https://www.mdpi.com/article/10.3390/foods10040708/s1, Figure S1: Area of apples by cropping density in hectares (%) in the Republic of Croatia for 2012 and 2017, Figure S2. Health benefits of apple, Table S1: Distribution of apple production in the Republic of Croatia for 2017 and 2018, Table S2: Area under cultivation, production and yield of apples by the main growing regions in the Republic of Croatia, Table S3: Total area of plantations by apple cultivar in hectares in the Republic of Croatia, Table S4: Some differences between modern cultivation and old apple cultivars growing in Croatia.
