# Supplementary material for: The Perspective of Croatian Old Apple Cultivars in Extensive Farming for the Production of Functional Foods
Source: Foods. 2021 Mar 26;10(4):708. doi: 10.3390/foods10040708 (PMC8065821; doi:10.3390/foods10040708)
Supplement: Supplementary file 1 [file foods-10-00708-s001.pdf]

Table S1. Distribution of apple production in the Republic of Croatia for 2017 and 2018.

Year	Type of production	Quantity (t)
2017	Total production	56 570
	Intensive production (for market)	55 790
	In extensive farming (mostly for own use)	780
2018	Total production	92 476
	Intensive production (for market)	88 716
	In extensive farming (mostly for own use)	3 760

Table S2. Area under cultivation, production and yield of apples by the main growing regions in the Republic of Croatia [1]

Geographical location	Year	Harvest area (ha)	Production (t)	Yield (t ha^{-1})
Republic of Croatia (total)	2010	6 599	89 124	13.5
	2011	6 553	99 676	15.2
	2012	5 980	37 414	6.3
	2013	5 377	121 738	22.6
	2014	5 944	96 703	16.3
	2015	5 756	96 182	16.7
	2016	5 890	44 176	7.5
	2017	4 838	55 790	11.5
	2018	4 728	90 254	19.1
	2019	4 946	68 352	13.8
Continental Croatia	2010	6 072	82 003	13.5
	2011	6 029	95 586	15.9
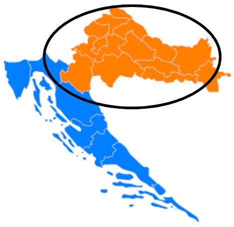	2012	5 502	36 485	6.6
	2013	5 109	120 054	23.5
	2014	5 649	93 421	16.5
	2015	5 397	92 885	17.2
	2016	5 537	38 811	7.0
	2017	4 551	54 428	12.0
	2018.	4 453	85 817	19.3
	2019	4 616	66 797	14.5
Adriatic Croatia	2010	527	7 121	13.5
	2011	524	4 090	7.8
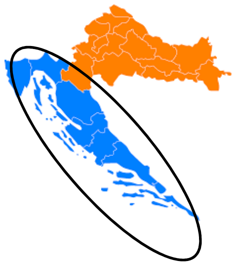	2012	478	929	1.9
	2013	268	1 684	6.3
	2014	295	3 282	11.1
	2015	359	3 297	9.2
	2016	353	5 365	15.2
	2017	287	1 362	4.7
	2018	275	4 437	16.1
	2019	330	1 555	4.7

Table S3. Total area of plantations by apple cultivar in hectares in the Republic of Croatia [1]

Apple cultivar	Harvest area (ha)		Share 2017	Change	
	2012	2017	In %	In ha	In %
Apples (total)	4798.64	4459.09	/	-339.55	-7.08
Boskoop	6.54	4.30	0.10	-2.24	-34.25
Braeburn	106.74	122.32	2.74	15.58	+14.60
Cox Orange	0.19	0.15	0.00	-0.04	-21.05
Cripps Pink	79.64	90.32	2.03	10.68	+13.41
Elstar	91.77	69.17	1.55	-22.60	-24.63
Florina	108.9	75.70	1.70	-33.20	-30.49
Fuji	50.33	119.72	2.68	69.39	+137.87
Gala	194.64	309.03	6.93	114.39	+58.77
Golden Delicious	665.58	701.40	15.73	35.82	+5.38
Granny Smith	275.7	245.02	5.49	-30.68	-11.13
Idared	1977.24	1612.26	36.16	-364.98	-18.46
Jonagold	456.86	431.12	9.67	-25.74	-5.63
Red Delicious	66.37	88.27	1.98	21.90	+33.00
Reinette	8.14	8.52	0.19	0.38	+4.67
Pinova	/	16.58	0.37	16.58	/

Table S4. Some differences between modern cultivation and old apple cultivars growing in Croatia

Growing practice	Modern apple cultivation	Old apple tree growing
Duration of cultivation in years	Up to 15-20	Up to 80-100
Rootstock	Low vigor (M9)	Very vigorous (seedlings)
Support system for fruit trees	Yes	No
Fertigation system	Yes	No
Spraying with pesticides	15-30	< 5
Anti-hail nets	Mostly Yes	No
Anti-frost system	Mostly Yes	No
Use of chemicals	Yes	No
Use of mechanization	Intensive	Extensive

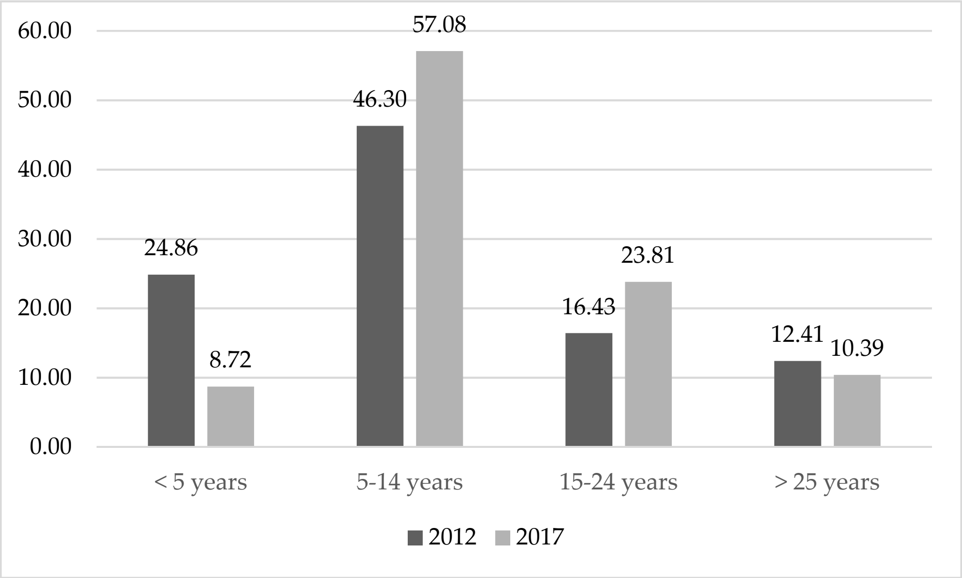

Figure S1. Area of apples by cropping density in hectares (%) in the Republic of Croatia for 2012 and 2017 [1]

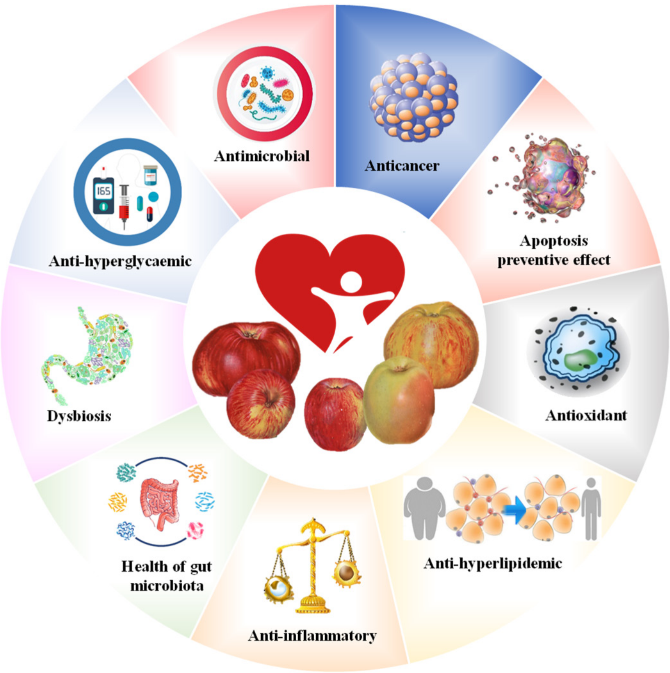

Figure S2. Health benefits of apple

References

1. Croatian Bureau of Statistics. Available online: https://www.dzs.hr/. Availabe online: (accessed on 05 September 2020).
